# A web-based microsatellite database for the Magnaporthe oryzae genome

**DOI:** 10.6026/97320630012388

**Published:** 2016-11-29

**Authors:** Pankaj Kumar Singh, Akshay Singh, Deepak V. Pawar, B. N. Devanna, Jyoti Singh, Vinay Sharma, Tilak R. Sharma

**Affiliations:** 1 National Research Centre on Plant Biotechnology, IARI, New Delhi 110012, India; 2 Department of Bioscience and Biotechnology, Banasthali University, Tonk, Rajasthan 304 022, India

**Keywords:** M. oryzae, SSRs, MMDB, rice blast

## Abstract

Microsatellites have been widely utilized for molecular marker development. Codominant and multiallelic nature of these simple repeats have several advantages over other types of molecular markers. Their broad applicability in the area of molecular biology like gene mapping, genome characterization, genome evolution, and gene regulation has been reported in various crop plants, animals and fungi. Considering these benefits of the SSR markers, a MMDB (Magnaporthe oryzae Microsatellite Database) was developed to help in understanding about the pathogen and its diversity at strains level of a particular geographic region, which can help us to make a proper utilization of blast resistance genes in the region. This microsatellite database is based on whole genome sequence of two M. oryzae isolates, RML-29 (2665 SSRs from 43037792 bp) and RP-2421 (3169 SSRs from 45510614 bp). Although, first M. oryzae genome (70-15) was sequenced in 2005, but this sequenced isolate is not a true field isolate of M. oryzae. Therefore, MMDB has great potential in the study of diversification and characterization of M. oryzae and other related fungi.

**Availability:**

http://14.139.229.199/home.aspx

## Background

Rice blast disease caused by a fungal pathogen Magnaporthe oryzae, which results up to 90% crop yield loss during severe epidemics [1]. Rice fulfils food energy requirement of 2/3rd of world’s population. Understanding about the pathogen and its diversity among different strains of a particular geographic region can help breeders for proper deployments of blast resistance genes in the region. DNA markers are the unique regions within a genome which may be associated with the genes responsible for specific trait in an organism. Microsatellite or simple sequence repeat markers are stretches of DNA in which the same short nucleotide sequence is tandemly repeated within the genome [2]. These sequences are also known as simple sequence repeats (SSRs) or simple tandem repeats (STRs). SSR is generally a 1-6 nucleotide sequence variations present across the eukaryotic genome [3,4, 5]. Polymorphism, or variation, among SSR markers is determined by the number of times of the base sequence repeats. Its hyper-variability in among the related organisms makes them excellent markers for phenotype mapping, marker assisted selection, genotype identification and analysis of genetic diversity. The nature of SSRs gives them a number of advantages over other molecular markers by their abundances in genome, high-level of polymorphisms, codominance nature, open accessibility, simple assay method, and feasibility to use at high-throughput level. They are one of the most advanced marker technologies, after single nucleotide polymorphism (SNP) markers, available in genetic research. Considering these above benefits, a SSR marker database was developed from genomes of two M. oryzae isolates, RML-29 (avirulent, designated name Mo-nwi-55) and RP-2421 (virulent, designated name Mo-nwi-31). These two isolates were selected for the database development on the basis of their virulence spectrum towards different rice blast resistance genes.

## Methodology

### Methodology of database development

A database of SSR markers identified in the M. oryzae genomes was developed and named as MMDB (Magnaporthe oryzae Microsatellite Database).The database was constructed with the help of Microsoft Visual Studio 2013 for designing web pages, which were programmed through ASP.NET framework 4.0 using C# programming language. Database tables were stored in Microsoft SQL Server 2008.

### Utility of the database (MMDB)

The database comprised of eight tabs, viz Home, About, Search, Analysis, Tutorial, Feedback, Team and Contact Us. Search option is further divided into two sub-tabs (for two strains) and each subtab is meant for searching microsatellites and their respective primers available in each genome. Tutorial has also been provided to describe the various steps used in the MMDB.

## Results

Genomes of two M. oryzae isolates were sequenced by using Illumina HiSeq-100 and Roche 454 platforms. The sequencing reads of both the genomes were assembled with the help of reference sequence 70-15 (version 8, www.broad.mit.edu). The reference genome (70-15) size is approximately 41 Mb and the genome comprises of 7 chromosomes. Besides that an extra short pseudo chromosome reported by some researchers in this fungus has been included [6]. We found about 3-4 Mb sized contigs in both the genomes after assembly which did not match with the reference genome. The unmatched contigs were considered as unique to the genome of respective isolate.

**Figure 1:**
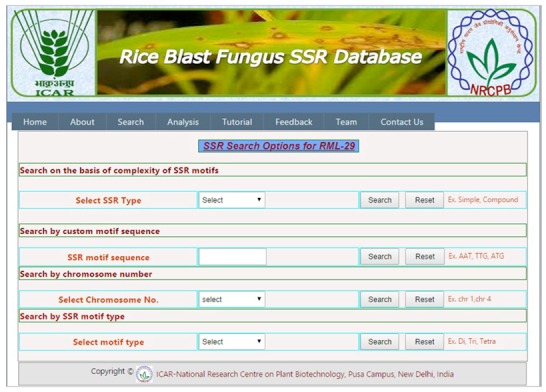
A snapshot of the database Analysis web page showing different search criteria in the website

All the assembled 7 chromosomes, an extra mini chromosome and the unmatched contigs were passed through MIcroSAtellite identification tool (MISA) to identify and find the location of perfect and compound microsatellites in two M. oryzae genomes. In this analysis, SSR motifs, repeat number, repeat types, length and size of the repeat, start and end position of the repeat, and their nucleotide sequences were obtained and their details were mentioned under Analysis tab of the MMDB. Repeat motifs, which were unable to yield primers, were excluded from this analysis. Overall 2665 SSRs were identified in RML-29 genome, including simple (2235) and compound (430) repeats, while more number of SSRs (3169) were obtained in the other strain RP-2421 of M. oryzae (Table 1). Types of SSR motifs were also categorized into different groups (Di to Hexa nucleotides) for both the strains of M. oryzae (Table 2). All SSR related information could be obtained by search tab of MMDB. Under search option, we can separately access microsatellite and SSR primers for each strain. In each strain specific search, different search criteria like search by SSR type, by SSR motif sequence, by chromosome number and by type of motif are provided (Figure 1).

## Conclusion

SSR markers developed in this analysis can be helpful for rice blast research community to explore diversity in M. oryzae population and study its evolution. Thus, the MMDB database has a great potential to study the diversification and characterization of rice blast fungus as well as other related fungi.

## Authors’ contribution

PKS and AS designed and developed the database under the guidance of TRS at ICAR-NRCPB, New Delhi. All authors contributed to the writing of the manuscript.

## Conflict of interests

The authors confirmed that this research article content has no conflict of interest.

## Figures and Tables

**Table 1:** List of total identified SSRs in Magnaporthe oryzae isolates

Isolate	Type	Number	Percentage
RML-29	Simple	2235	83.86
	Compound	430	16.14
	Total	2665	100
RP-2421	Simple	2287	72.83
	Compound	882	27.83
	Total	3169	100

**Table 2:** Type of SSR motifs identified in Magnaporthe oryzae isolates

Isolate	Motif type	Number	Percentage
RML-29	Di	635	27.2
	Tri	1429	61.2
	Tetra	202	8.65
	Penta	32	1.37
	Hexa	37	1.58
RP-2421	Di	603	26.34
	Tri	1422	62.13
	Tetra	196	8.65
	Penta	30	1.31
	Hexa	36	1.57
